# The Usefulness of Testosterone in Saliva Tests to Detect Testosterone Deficiency in Men with Advanced Chronic Kidney Disease: A Single-Center Study

**DOI:** 10.3390/jcm14082818

**Published:** 2025-04-19

**Authors:** Ksymena Leśniak, Arkadiusz Lubas, Stanisław Niemczyk

**Affiliations:** Department of Internal Diseases, Nephrology and Dialysis, Military Institute of Medicine—National Research Institute, Szaserów Street 128, 04-141 Warsaw, Poland; alubas@wim.mil.pl (A.L.); sniemczyk@wim.mil.pl (S.N.)

**Keywords:** advanced chronic kidney disease, dialysis, saliva testosterone, male testosterone deficiency, serum testosterone

## Abstract

**Background:** Hypogonadism frequently occurs among men with chronic kidney disease (CKD) and is a highly unfavorable prognostic factor. Therefore, a simple and common screening for testosterone deficiency may be important. The measurement of testosterone in saliva appears to be an attractive alternative to serum testosterone. This study aimed to assess the usefulness of determining free testosterone concentration in saliva to detect testosterone deficiency in men with advanced CKD, including those on dialysis. **Methods:** A total of 77 adult, male patients (aged 41–89 years old)—30 with CKD stage G3-G4, 30 on hemodialysis (HD), and 17 on peritoneal dialysis (PD)—were evaluated. The concentration of free testosterone was determined in saliva (SalFT), while the concentration of total testosterone (TT) was determined in blood serum. Serum-free testosterone levels were calculated (cFT). **Results:** SalFT did not differ from cFT in the CKD (*p* = 0.547) and PD groups (*p* = 0.409). In the HD group, SalFT was higher than cFT (*p* = 0.009). SalFT was positively correlated with cFT (r = 0.435 in the CKD and r = 0.479 in the HD) and TT (r = 0.451 in CKD), but only in the group of patients with SalFT levels below 140 pg/mL and 120 pg/mL, respectively. A cut-off value of SalFT ≤ 60.6 pg/mL showed 73.9% sensitivity and 77.8% specificity for testosterone deficiency recognition. **Conclusions:** Our study supports the value of SalFT measurement as a non-invasive approach in the diagnosis of testosterone deficiency in men with advanced CKD, as well as patients on hemodialysis.

## 1. Introduction

The kidneys participate at many levels in the metabolism of various hormones, including sex hormones, and endocrinological disorders develop early in the course of chronic kidney disease (CKD), increasing their intensity together with its progression. Furthermore, such conditions as malnourishment, inflammation, comorbidities, commonly used drugs, or metabolic acidosis may contribute to the development and intensification of hormonal disorders in CKD [1]. The most frequent gonadal disorder in males with CKD is hypogonadism, i.e., a syndrome of clinical symptoms and signs closely associated with testosterone deficiency. In males with CKD, both primary (Leydig cell dysfunction) and secondary (suppression of the hypothalamo–pituitary–testicular axis) hypogonadism occur, which are caused by functional disorders of a multifactorial origin [1,2,3]. The incidence of hypogonadism increases along with the progression of renal failure (from 17% in CKD stage 1 to 57% of the patients in CKD stage 5 without dialysis and up to 60% in hemodialysis patients), accounting for a much higher percentage compared with the general population [4,5,6,7]. In spite of the abundant literature on hemodialysis patients, still few reports are available concerning patients treated with peritoneal dialysis [8,9].

It is well known that testosterone deficiency is associated not only with sexual disorders but also with an unfavorable cardiometabolic profile and an increase in mortality for patients with renal failure [9,10,11,12,13,14,15,16]. Considering the fact that testosterone supplementation is possible and its safety in CKD patients, including those on dialysis, has been demonstrated in several clinical studies, it is important to identify a group of men with hypogonadism [17].

The determination of total testosterone concentration in blood serum is used as a screening test for the diagnosis of hypogonadism; however, if a disorder related to sex hormone-binding globulin (SHBG) is suspected or the measured total testosterone concentration is low (200–400 ng/dL), it is recommended to determine the concentration of free testosterone, especially necessary in patients with CKD [18].

Much controversy raises the specification of the lower limit of the normal range for testosterone, both free and total. These standards vary depending on the recommendations, due to the fact that research has been carried out in different populations with the use of different laboratory methods for assessing this hormone concentration [18,19,20,21]. Serum TT and FT concentrations can be determined by direct and indirect methods. In hypogonadism, due to the low levels of these hormones (especially in severe forms), their assessment has always been a challenge [22].

Over the years, methods for the determination of total testosterone in blood serum have been improved: from the first indirect radioimmunological methods (radioimmunoassay; RIA) introduced about 40 years ago, through ready-made commercial kits based on direct methods (automated immunoassay platform), to the current gold standard, i.e., indirect tests using mass spectrometry [22,23,24]. Equilibrium dialysis is the reference method for the measurement of free testosterone in serum. However, it has many drawbacks limiting its use in everyday practice (it is labor-intensive, time-consuming, and expensive); therefore, clinicians use mathematical equations to estimate the concentration of free testosterone, which is consistent with the recommendations. The disadvantage of the calculation method is the dependence of the result on the level of serum TT and protein concentrations (SHBG, albumin). Moreover, these formulas have not been validated in patients with advanced kidney disease and on dialysis [18,25].

It seems that the determination of testosterone concentration in saliva can be an alternative method of determining its concentration [26,27]. Saliva is a recognized material for steroid analysis, and its collection is a non-invasive alternative to plasma and serum. Saliva collection is a fairly simple method that allows the patient to obtain diagnostic material on his own in a convenient way and submit it for analysis. Compounds that are determined in saliva are characterized by high stability; however, the diagnostic value of testosterone tests in saliva is affected by rapid fluctuations in the concentration of these steroids in saliva. Repeated samples are required to obtain reliable information [28].

Salivary testosterone is a measure of the concentration of the free fraction of testosterone in plasma that passively diffuses across salivary gland cell membranes, regardless of the rate of salivary secretion, and therefore salivary testosterone is expected to provide a better measure of testosterone bioavailability in the body than serum testosterone [26,29].

The available body of literature provides data regarding the effectiveness of laboratory methods, which show a very high correlation between the concentration of free and total testosterone in saliva and plasma (0.97 and 0.83, respectively) [30,31]. Many studies, but not all, show a highly significant correlation between salivary testosterone and free serum testosterone in adult men in the general population [32,33,34,35,36]. In previous studies, the usefulness of salivary testosterone measurement in patients with chronic kidney disease was not defined; however, recently, Cardoso et al. presented that saliva could be used as a valuable tool for assessing the androgen profile in patients with end-stage renal disease on hemodialysis [37,38,39]. However, there is still a lack of data on patients with CKD, both in the pre-dialysis period and on dialysis, including peritoneal dialysis.

This study aimed to assess the usefulness of determining the concentration of free testosterone in saliva in detecting testosterone deficiency in men with CKD stage G3–G4, as well as patients on hemodialysis or peritoneal dialysis.

## 2. Materials and Methods

Seventy-seven adult, male consecutive patients (aged 41–89 years old)—thirty with chronic kidney disease stage G3-G4 [eGFR < 60 mL/min/1.73 m^2^ and ≥15 mL/min/1.73 m^2^ according to the Kidney Disease: Improving Global Outcomes (KDIGO) definition], thirty on hemodialysis, and seventeen on peritoneal dialysis—who were admitted to the nephrology and dialysis center and who signed informed consent were evaluated. From the group of dialysis patients, we included only those who had been on dialysis for at least 3 months. Hemodialysis patients had HD sessions 3 times a week. Peritoneal dialysis patients were treated with continuous ambulatory peritoneal dialysis (CAPD). In the study groups, the testosterone profile was assessed.

Patients with active cancer, advanced and uncontrolled heart or liver failure, recurrent inflammation, a severe form of secondary parathyroidism, and treatment of endocrine diseases (including testosterone therapy) were excluded from the study.

The local ethics committee accepted the study protocol (Military Institute of Medicine Bioethics Committee: approval number 50/WIM/2016). The study was performed in accordance with principles outlined in the Declaration of Helsinki. Written informed consent was obtained from all study participants.

In the studied groups of patients, the concentration of free testosterone was determined in saliva (SalFT), while in blood serum, the concentration of total testosterone (TT), luteinizing hormone (LH), prolactin (PRL), sex hormone-binding globulin (SHBG), creatinine, and albumin was determined.

Additionally, serum-free testosterone levels were calculated (cFT) from total testosterone, SHBG, and albumin using The International Society for the Study of the Aging Male (ISSAM) calculator:” http://www.issam.ch (accessed on 15 January 2024)” [25].

eGFR was obtained using the Modification of Diet in Renal Disease formula (MDRD) [40].

Blood samples for testing were collected in the morning between 7:00 a.m. and 11:00 a.m., while in the case of hemodialysis patients, they were collected directly before the dialysis procedure. Immediately after collection, blood samples were sent to the Department of Laboratory Diagnostics for testing, while blood samples intended for testing in the Laboratory Department of the Clinic of Endocrinology and Internal Diseases were centrifuged after collection, and the serum was frozen at −70 °C until assays.

Saliva samples were collected simultaneously with blood samples between 7:00 a.m. and 11:00 a.m. and before dialysis sessions in hemodialysis patients. Patients delivered at least 0.5 mL of saliva by spitting directly into glass tubes intended for saliva collection, which were then frozen at −20 °C until assays. Patients were informed that eating, drinking, chewing gum, or brushing teeth should be avoided for 30 min before sampling.

The following tests were used for laboratory determinations of selected parameters:

Testosterone in saliva—ELISAkit (DRG, Marburg, Germany); serum total testosterone, LH, SHBG, and PRL-electrochemiluminescence method (Roche Elecsys analyzer, Mannheim, Germany); serum creatinine–enzymatic method (Roche Diagnostics, Warsaw, Poland), and serum albumin–colorimetric method (Roche Diagnostics, Warsaw, Poland).

As there are no consistent guidelines providing the cut-off value for TT and cFT for the diagnosis of hypogonadism [3,18,19], the following was adopted in the presented study:

Total testosterone deficiency was considered if serum level was between <2.88 ng/mL= 10 nmol/L (normal range in a local laboratory: 2.8–8.2 ng/mL). Free testosterone deficiency was considered if the calculated free testosterone concentration was between <50 pg/mL and <0.17 nmol/L [41].

### Statistical Analysis

The quantitative variables were presented as the mean, standard deviation, median, and range. The consistency with the normal distribution was checked using the Shapiro–Wilk test. The categorical data were presented as numbers and occurrences. Differences between two quantitative variables were tested using the t-student test for dependent data. Differences between more than two groups were checked using Analysis of Variance (ANOVA) if the conditions of normal distribution and variance equality were met; otherwise, the Kruskal–Wallis test was conducted. A comparison of categorical data was performed using the chi-squared test. For correlation analysis, the Pearson correlation coefficient or Spearman correlation coefficient was determined according to normal distribution criteria fulfillment. ROC analysis was performed to identify the optimal cut-off value. In all tests, *p* < 0.05 was considered statistically significant. Statistical analysis was performed using the Tibco Statistica v. 13.3 (TIBCO Software Inc., Greenwood Village, CO, USA) package.

## 3. Results

The study included seventy-seven male patients (age 63.2 ±10.7 years): thirty with chronic kidney disease stage G3-G4 (CKD group), thirty with kidney failure treated with hemodialysis (HD group), and seventeen with kidney failure treated with peritoneal dialysis (PD group). In the CKD group, the mean creatinine concentration was 2.29 ± 0.81 mg/dL, and the mean estimated glomerular filtration rate (eGFR) was 32.6 ± 10.6 mL/min/1.73 m^2^. Table 1 summarizes selected clinical, biochemical, and hormonal parameters of the studied patient groups (Table 1).

The testosterone concentration in saliva did not significantly differ from the calculated free testosterone in blood serum in the CKD (*p* = 0.547) and PD (*p* = 0.409) groups. However, in the HD group, a significantly higher concentration of testosterone in saliva was observed compared to calculated free testosterone in serum (*p* = 0.009).

Initially, in all considered groups, concentrations of SalFT did not correlate with total and calculated testosterone concentrations (Table 2).

**Figure 1:**
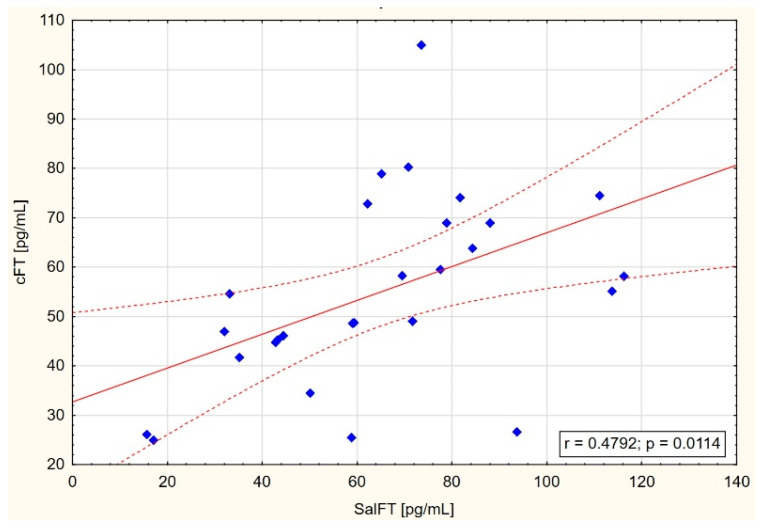
Scatter graph with correlation between SalFT < 140 pg/mL and cFT in HD group.

**Figure 2:**
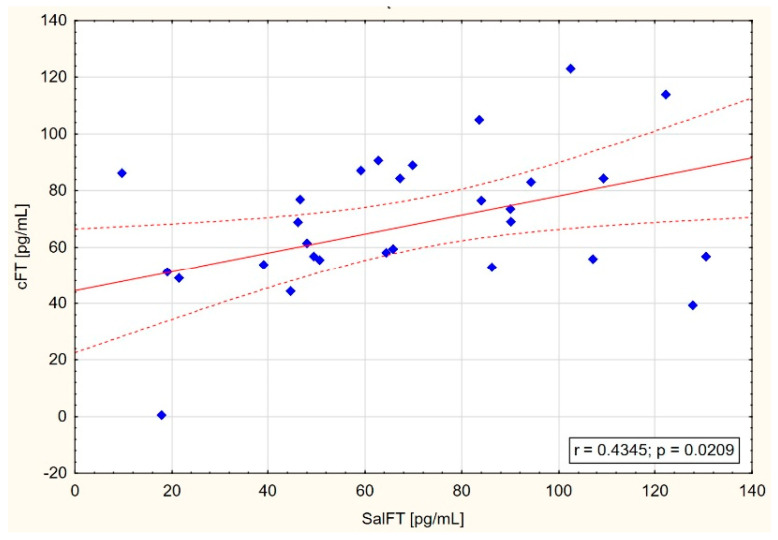
Scatter graph with correlation between SalFT < 140 pg/mL and cFT in CKD group.

**Figure 3:**
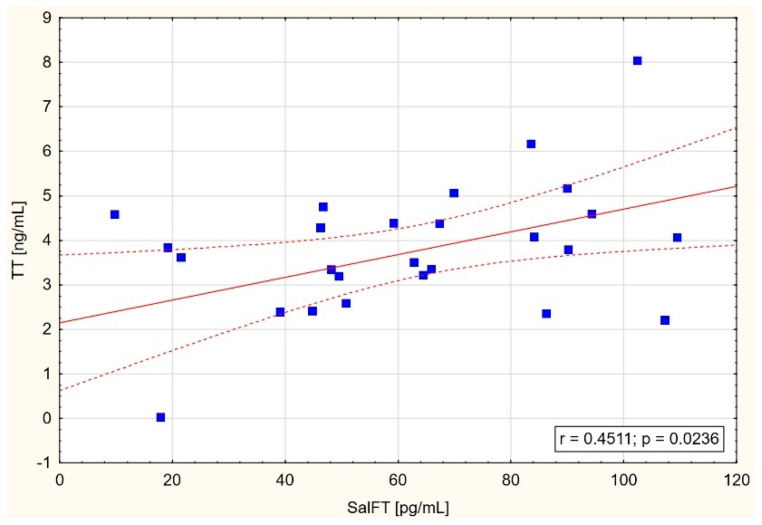
Scatter graph with correlation between SalFT < 120 pg/mL and TT in CKD group.

On the other hand, narrowing maximal results of testosterone concentration in saliva to 140 pg/mL or even 120 pg/mL showed increasing correlations between SalFT and cFT and TT concentrations, especially in CKD and HD groups (Table 3, Figure 1, Figure 2 and Figure 3).

**Figure 4:**
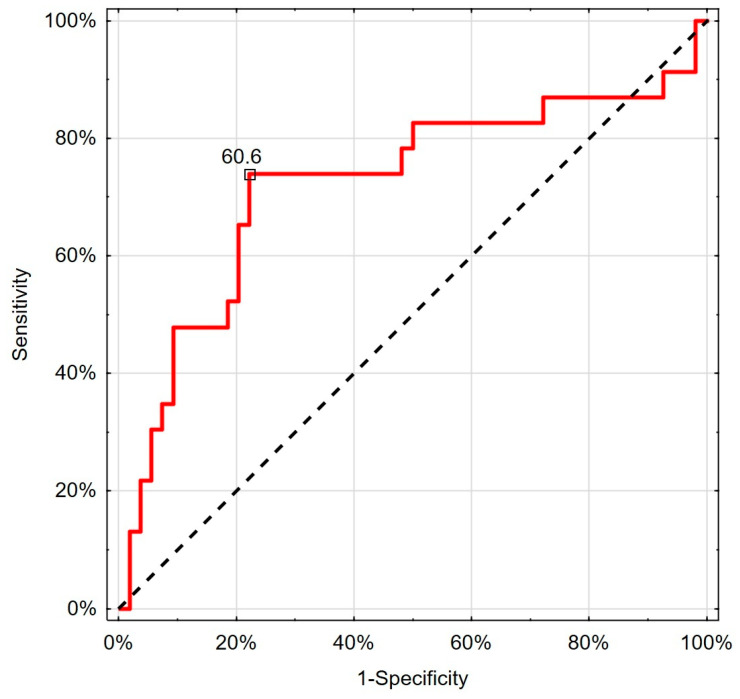
Visual presentation of ROC analysis for salivary testosterone in predicting cFT deficiency.

Regarding TT and cFT limit values for testosterone deficiency recognition, ROC analysis revealed that the SalFT cut-off value below or equal to 60.6 pg/mL can be helpful in the screening of hypogonadism in chronic kidney disease patients (Table 4, Figure 4).

Using the SalFT cut-off value ≤ 60.6 pg/mL, testosterone deficiency could be identified in 29 patients (12 CKD, 12 HD, 5 PD). A comparison of SalFT effectiveness with other referenced methods of testosterone deficiency recognition is presented in Table 5.

## 4. Discussion

In the present study, positive correlations between free testosterone in saliva and both free calculated testosterone in blood serum (in the CKD group: r = 0.435 and *p* = 0.021; in the HD group: r = 0.479 and *p* = 0.011) and total serum testosterone (in the CKD group: r = 0.451 and *p* = 0.024) were found, but only in the group of patients with SalFT levels below 140 pg/mL or even 120 pg/mL (Figure 1, Figure 2 and Figure 3). These results may indicate the usefulness of measuring testosterone in saliva in detecting testosterone deficiency among men with CKD stage G3–G4 as well as patients on hemodialysis.

Medical journals have published many studies of men in the general population in whom positive correlations between SalFT and FT in blood serum were reported [33,34,35]. For example, Goncharov et al. demonstrated that the concentration of free testosterone in saliva in morning samples correlated with the calculated free testosterone in the blood, both in healthy men (r = 0.754, *p* = 0.001) and in patients with androgen deficiency (r = 0.889, *p* = 0.0001) [33]. Morley et al. observed that salivary testosterone correlated with total testosterone in blood serum (r = 0.346), bioavailable testosterone in blood serum (r = 0.633), and free calculated testosterone in blood serum (r = 0.451) [34]. In turn, Arregger et al. revealed that salivary testosterone correlated positively with all circulating androgens; however, the strongest correlation was observed with free testosterone in both men with normal gonadal function (r = 0.92) and those with hypogonadism (r = 0.97) [35].

On the other hand, Fiers et al. did not report any correlation between the concentration of testosterone in saliva and the concentration of free testosterone in blood serum determined by equilibrium dialysis [36].

Comparing our results with the literature data, in the study of Cardoso et al. enrolling 60 hemodialysis patients with reduced libido, the authors also reported positive correlations between testosterone in saliva and free calculated testosterone in serum; however, in their study, higher correlation coefficients were demonstrated: r = 0.95 (*p* = 0.0001) in dialysis patients and r = 0.97 (*p* = 0.0001) in the control group of men without renal failure. Moreover, testosterone in saliva positively correlated with total serum testosterone (r = 0.80) and bioavailable testosterone (r = 0.76). The differences between our study and that of Cardoso et al. can be the result of different inclusion criteria because our group consisted of consecutive patients, not only with hypogonadism symptoms. Moreover, discrepancies may come from the other methods of testosterone measurement and different sizes of study groups [39].

It is worth mentioning that the testosterone concentrations in saliva reported in our study were similar to free testosterone concentrations calculated in blood serum, especially in the CKD and PD groups, but not in the HD group (Table 1). In the present study, the mean concentration of SalFT in the HD group was 74.3 pg/mL, while cFT was 54.4 pg/mL, respectively. Goncharov et al. also reported higher salivary concentrations than calculated serum-free testosterone in men with very low testosterone levels [33]. In the interpretation of our results, the fact that salivary free testosterone is a direct measurement, while in blood serum, it is only calculated on the basis of specific parameters, is important. Based on our study, it is not possible to determine which of these methods is more appropriate, and further research is needed.

Significant difficulties are encountered trying to define testosterone deficiency based on free testosterone concentration in blood serum because not all guidelines provide a cut-off value for FT. The lower limits of the normal range for serum FT were recorded in only a few such documents and ranged from 173 to 347 pmol/L (50 to 100 pg/mL) [19,41,42,43,44].

For example, in our study, testosterone deficiency based on FT values affected 50% of men on hemodialysis, while in the study by De Silva et al., as many as 75.5% (104/136) men on hemodialysis were affected with a similar mean age to our group [45]. One of the reasons for these discrepancies may be the different FT cut-off value adopted for the definition of hypogonadism, because in our study we assumed a strict level of FT< 50 pg/mL, while in the study performed in the United Kingdom, the level of FT < 220 pmol/L = 63.5 pg/mL was adopted [45].

Regarding TT and cFT limit values for testosterone deficiency recognition, we decided to assess the frequency of testosterone deficiency by measuring the free testosterone fraction in saliva. In the present study, the ROC analysis revealed that the SalFT cut-off value below or equal to 60.6 pg/mL can be helpful in the screening of hypogonadism in chronic kidney disease patients (Table 4, Figure 4). The value of testosterone deficiency defined in this way was similar to the frequency defined according to the concentration of total testosterone in serum (37.7% vs. 35.1%; *p* = 0.838) (Table 5).

It is worth noting that our SalFT cut-off value (≤0.210 nmol/L; ≤60.6 pg/mL) is close to those recently proposed. Arregger et al. suggested the testosterone measurement in saliva as a biomarker for the diagnosis of male androgen deficiency with the cut-off for Sal-T ≤ 0.195 nmol/L [35]. Similarly, Goncharov et al. demonstrated the mean salivary testosterone concentration was 0.215 nmol/L in men with androgen deficiency [33].

In a study performed in Argentina, the percentage of hemodialysis patients with hypogonadism diagnosed based on Sal-T concentration (below 0.129 nmol/L) was 22%, which is much lower than in our study (40%) [39]. At the same time, it is worth mentioning that the age of the studied populations differed significantly and was higher in our study. The median age of hemodialysis patients was 61.2 ± 10.1 years, while in the study conducted by Cardoso et al., the median age was 39.5 (range 18–60 years) [39].

Although we could not find a significant correlation between cFT and SalFT in PD patients, the frequency of hypogonadism, recognized based on ROC analysis, was similar to that using the cFT threshold. In this calculation, the results do not differ from those measured in HD patients (Table 5). On the other hand, SalFT correlated with cFT in CKD patients, but ROC analysis was inconclusive, and the proposed SalFT cut-off value (similar to PD and HD groups) recognized many more cases of hypogonadism than using the cFT threshold. This suggests that the SalFT threshold value can be higher for hypogonadism recognition in the CKD group. However, further studies are needed to elucidate this problem.

Interestingly, in the present study, the percentage of patients with testosterone deficiency in the PD group is lower than in the HD group (Table 5). 

In the study involving 79 patients (43 on hemodialysis and 36 on peritoneal dialysis), Cigarrán et al. also observed a difference in the incidence of testosterone deficiency between the patients treated with various dialysis methods. The authors found a testosterone deficiency in the serum of 39.5% of the males receiving hemodialysis treatment compared with just 5.6% of the patients on peritoneal dialysis. The authors seek the causes of the differences in a possibly lower loss of testosterone and higher protein loss during peritoneal dialysis compared with the hemodialysis procedure, which may result in an increase in the free testosterone pool. However, further studies are needed concerning the elimination of hormones during dialysis procedures [8].

We assume that the assessment of salivary testosterone in the group of patients on peritoneal dialysis may be as useful as the measurement of total and free testosterone in blood serum in detecting testosterone deficiency, but still, it requires further research.

An additional advantage of our study was a broader assessment of hormone levels, including gonadotropin (LH) and prolactin (PRL), to enable a better understanding of the mechanism of hypogonadism in the studied groups of patients. So, we found increased mean serum LH concentrations in all groups, while in the groups of dialysis patients, increased mean PRL concentration values were additionally detected, which was in concordance with the available literature reports (Table 1).

It has been speculated that the factors responsible for the abnormalities in the synthesis of androgens in uremia may include a reduction of the frequency of GnRH pulses or an impairment of hypophyseal response [46]. An inhibitor of the receptor of circulating LH contributes to peripheral resistance of the Leydig cells and to an impairment of the feedback mechanism [47]. While LH concentration is increased due to reduced excretion with urine in males with CKD, the levels of bioactive LH forms are reduced, contributing to testosterone deficiency [48]. In addition, an overproduction of prolactin and its reduced clearance in patients with CKD lead to hyperprolactinemia, resulting in an inhibition both of the pulsatile GnRH secretion and of the mechanism of positive feedback response of estrogens to gonadotropin secretion [49].

Although promising results, our study has some limitations. First, limited patient numbers in the studied groups can influence correlation significance and difference detection. For this reason, we did not test the differences in testosterone concentrations between CKD G3 and CKD G4 groups. Second, differences in age and treatment methods between CKD dialyzed and non-dialyzed patients can affect the SalFT threshold value for hypogonadism recognition. On the other hand, significant correlations and the possibility for hypogonadism recognition using salivary testosterone in men with advanced chronic kidney disease shown in our study are promising and indicate the need for further studies on larger patient groups.

## 5. Conclusions

The determination of free testosterone concentration in saliva seems to be a useful tool for the assessment of testosterone deficiency in men with advanced CKD as well as patients on hemodialysis. The non-invasive nature of the method may increase its availability, enable long-term testosterone level evaluation disorders, and thus personalize the treatment of patients with renal failure.

## Figures and Tables

**Table 1:** Selected clinical, biochemical, and hormonal parameters in the studied groups of patients.

		CKD	HD	PD	Significance *p*
		*n* = 30	*n* = 30	*n* = 17	
Age (years)	Mean ± SD	67.6 ± 9.2	61.2 ± 10.1	59.2 ± 12.2	0.014 *
	Median (min–max)	68.5 (45–89)	63 (42–84)	58 (41–77)	
BMI (mg/m^2^)	Mean ± SD	27.9 ± 4.7	28.9 ± 5.0	27.3 ± 4.2	0.508 *
	Median (min–max)	27.3 (19.7–40.1)	28.4 (18.9–38.8)	27.3 (18.9–35.1)	
Albumin (g/dL)	Mean ± SD	4.4 ± 0.3	4.1 ± 0.4	3.8 ±0.4	<0.001 *
	Median (min–max)	4.5 (3.9–4.9)	4.1 (3.2–4.7)	3.9 (2.5–4.2)	
Duration of dialysis (years)	Mean ± SD	-	2.75 ± 2.69	1.7 ± 1.6	0.010
	Median (min–max)	-	(0.3–12.8)	(0.3–6.8)	
TT (ng/mL)	Mean ± SD	3.8 ± 1.4	3.1 ± 1.3	3.7 ± 1.3	0.025
	Median (min–max)	3.9 (0.03–8.1)	2.7 (1.1–8.0)	3.6 (1.7–6.4)	
cFT (pg/mL)	Mean ± SD	68.6 ± 24.3	54.4 ± 18.6	69.5 ± 23.2	0.022 *
	Median (min–max)	65.1 (0.5–123.0)	51.8 (24.9–105.0)	67.8 (40.9–133.0)	
SalFT (pg/mL)	Mean ± SD	73.2 ± 39.1	74.3 ± 39.0	76.5 ± 29.7	0.959 *
	Median (min–max)	66.6 (9.7–186)	70.2 (15.6–177.6)	68.7 (35–149.7)	
SHBG (ug/mL)	Mean ± SD	4.6 ± 1.4	5.0 ± 3.3	4.6 ± 1.7	0.707
	Median (min–max)	4.3 (2–7)	4.2 (2.0–18.9)	4.9 (1.6–8.0)	
LH (IU/l)	Mean ± SD	11.7 ± 8.9	15.6 ± 16.7	18.5 ± 14.7	0.197
	Median (min–max)	9.1 (0.1–48.1)	9.8 (0.4–79.4)	12.8 (5.8–57.5)	
PRL (ng/mL)	Mean ± SD	10.8 ± 5.5	27.2 ± 20.3	22.4 ± 11.3	<0.001
	Median (min–max)	9.3 (4.2–31.2)	19.1 (9.4–103.8)	17.1 (13.3–57.0)	

* ANOVA test.

**Table 2:** Correlations between SalFT concentrations and age, serum total testosterone, and calculated free testosterone concentrations in the studied patient groups.

		SalFT		
		CKD	HD	PD
Age	r	−0.005	0.010	−0.298
	*p*	0.979	0.957	0.245
TT	r	0.257	0.284	−0.043
	*p*	0.171	0.128	0.867
cFT	r	0.233	**0.235**	0.190
	*p*	0.215	0.210	0.466

**Table 3:** Correlations between limited Sal-T concentrations results, serum total testosterone, and calculated free testosterone concentrations.

		SalFT					
		<140 pg/mL			<120 pg/mL		
		CKD	HD	PD	CKD	HD	PD
TT	r	0.352	0.315	−0.086	0.451	0.315	0.180
	*p*	0.066	0.110	0.753	0.024	0.110	0.521
cFT	r	0.435	0.479	0.170	0.610	0.479	0.332
	*p*	0.021	0.011	0.529	0.001	0.011	0.226

**Table 4:** Results of salivary testosterone ROC analysis for testosterone deficiency prediction.

Reference	Group	SalFT Cut-Off Value	Sensitivity [%]	Specificity [%]	AUC	Significance—*p*
TT < 2.88 (ng/mL)	CKD	59.2	57.1	65.2	0.571	0.605
	HD	59.4	62.5	85.7	0.674	0.094
	PD	54.2	50.0	84.6	0.538	0.852
	All	59.4	59.3	76.0	0.607	0.141
cFT < 50 (pg/mL)	CKD	59.2	50.0	60.7	0.607	0.527
	HD	59.4	73.3	93.3	0.760	0.009
	PD	60.6	75.0	84.6	0.788	0.023
	All	60.6	73.9	77.8	0.721	0.002

**Table 5:** Comparison of testosterone deficiency recognition in the studied groups.

Group	TT < 2.88 (ng/mL)	cFT < 50.0 (pg/mL)	SalFT ≤ 60.6 (pg/mL)	Significance—*p* TT: SalFT	Significance—*p* cFT: SalFT
CKD (*n* = 30)	7 (23.3%)	4 (13.3%)	12 (40.0%)	0.228	0.027
HD (*n* = 30)	16 (53.3%)	15 (50%)	12 (40.0%)	0.289	0.371
PD (*n* = 17)	4 (23.5%)	4 (23.5%)	5 (29.4%)	1.000	1.000
All (*n* = 77)	27 (35.1%)	23 (29.9%)	29 (37.7%)	0.838	0.239

## Data Availability

Data are available from the corresponding author upon reasonable request.
